# Application of PDT for Uterine Cervical Cancer

**DOI:** 10.1155/DTE.5.183

**Published:** 1999

**Authors:** T. Muroya, K. Kawasaki, Y. Suehiro, T. Kunugi, K. Umayahara, T. Akiya, H. Iwabuchi, H. Sakunaga, M. Sakamoto, T. Sugishita, Y. Tenjin

**Affiliations:** Department of Gynecology Sasaki Institute Kyoundo Hospital 1-8 Kanda, Surugadai, Chiyoda-ku Tokyo 101-0062 Japan

## Abstract

We have been performing PDT using Excimer Dye Laser (EDL) or YAG-OPO laser, a type of low power laser, both of which have a considerably higher degree of tissue penetration even when compared to PDT using Argon Dye Laser (ADL).

PDT is a relatively simple procedure without any bleeding and does not require anesthesia since it causes no pain. PDT is performed 48 h after intravenous injection of 1.5–2.0 mg/kg of PHE (Photofrin^®^). Precise spot irradiation is possible using a colposcope with an optical laser path. We also use a cervical probe which enables photoirradiation of the entire cervical canal.

We have performed PDT on 131 cases (95 CIS, 31 dysplasia, 1 vulval dysplasia (VIN), 3 squamous cell carcinoma, microinvasion, and 1 CIS + endocervical adenocarcinoma, microinvasion). Of these cases, 127 became CR (96.9%). The first CR case was 10 years ago and no recurrence has been observed yet.

PDT is extremely effective to preserve fertility. Except for sensitive reactions to sunlight, there are no noticeable side effects or difficulties related to pregnancy or delivery. We expect that in the near future PDT will be performed using diode lasers and without hospitalization due to new photosensitizers which have shorter retention times.


*Diagnostic and Therapeutic Endoscopy*, Vol. 5, pp. 183–190
Reprints available directly from the publisher
Photocopying permitted by license only

© 1999 OPA (Overseas Publishers Association) N.V.
Published by license under
the Harwood Academic Publishers imprint,
part of The Gordon and Breach Publishing Group.
Printed in Malaysia.

### Application of PDT for Uterine Cervical Cancer

T. MUROYA*, K. KAWASAKI, Y. SUEHIRO, T. KUNUGI, K. UMAYAHARA, T. AKIYA, H. IWABUCHI, H. SAKUNAGA, M. SAKAMOTO, T. SUGISHITA and Y. TENJIN

*Department of Gynecology, Sasaki Institute, Kyoundo Hospital, 1-8 Kanda, Surugadai, Chiyoda-ku, Tokyo 101-0062, Japan*

**We have been performing PDT using Excimer Dye Laser (EDL) or YAG-OPO laser, a type of low power laser, both of which have a considerably higher degree of tissue penetration even when compared to PDT using Argon Dye Laser (ADL).**

**PDT is a relatively simple procedure without any bleeding and does not require anesthesia since it causes no pain. PDT is performed 48 h after intravenous injection of 1.5–2.0 mg/kg of PHE (Photofrin®). Precise spot irradiation is possible using a colposcope with an optical laser path. We also use a cervical probe which enables photoirradiation of the entire cervical canal.**

**We have performed PDT on 131 cases (95 CIS, 31 dysplasia, 1 vulval dysplasia (VIN), 3 squamous cell carcinoma, microinvasion, and 1 CIS + endocervical adenocarcinoma, microinvasion). Of these cases, 127 became CR (96.9%). The first CR case was 10 years ago and no recurrence has been observed yet.**

**PDT is extremely effective to preserve fertility. Except for sensitive reactions to sunlight, there are no noticeable side effects or difficulties related to pregnancy or delivery. We expect that in the near future PDT will be performed using diode lasers and without hospitalization due to new photosensitizers which have shorter retention times.**

*Keywords:* Excimer dye laser, Photodynamic therapy (PDT), Photofrin® (PHE), Uterine cervical cancer, YAG-OPO laser

### INTRODUCTION

The discovery rates of dysplasia and CIS for cervical cancer has increased due to the recent awareness about and availability of cervical cancer examinations. As a result, there has been an increase in the number of patients and a decrease in the average age of patients. For these patients, it is desirable to perform operations to preserve fertility with respect to the quality of life of each patient.

As an operation to preserve fertility, conventional conization by cold knife (Sturmdorf operation) has traditionally been employed, but recent laser therapy using high-power lasers such as $CO_2$ laser, YAG laser and LEEP (Loop Electrosurgical Excision Procedure) are now frequently administered. It should be noted that these treatments are sometimes accompanied by unexpected bleeding, and since the treatment is accompanied by pain, anesthesia is necessary in most cases. In addition, a large portion

* Corresponding author. Tel.: (81-3) 3292-2052. Fax: (81-3) 3292-3376. E-mail: tmuroya@coral.ocn.ne.jp.

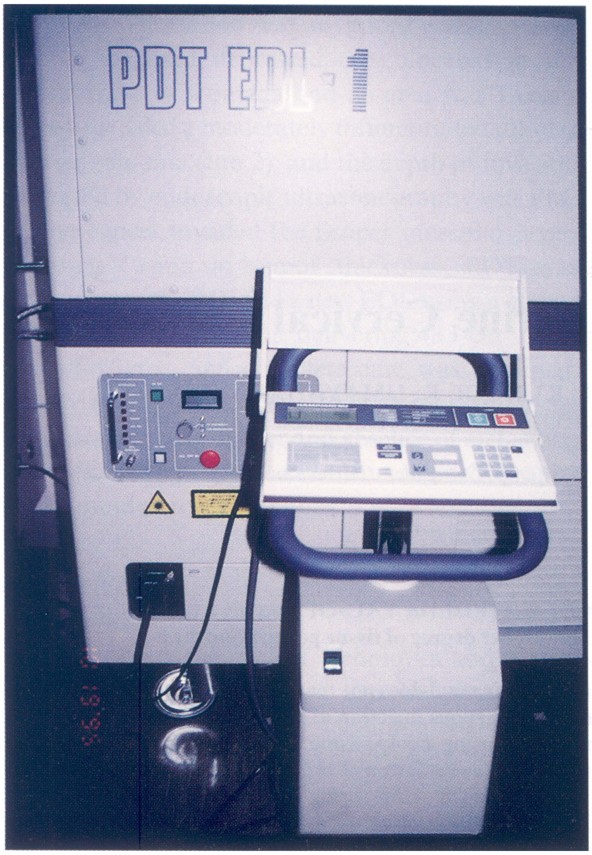

FIGURE 1 Excimer dye laser (EDL).

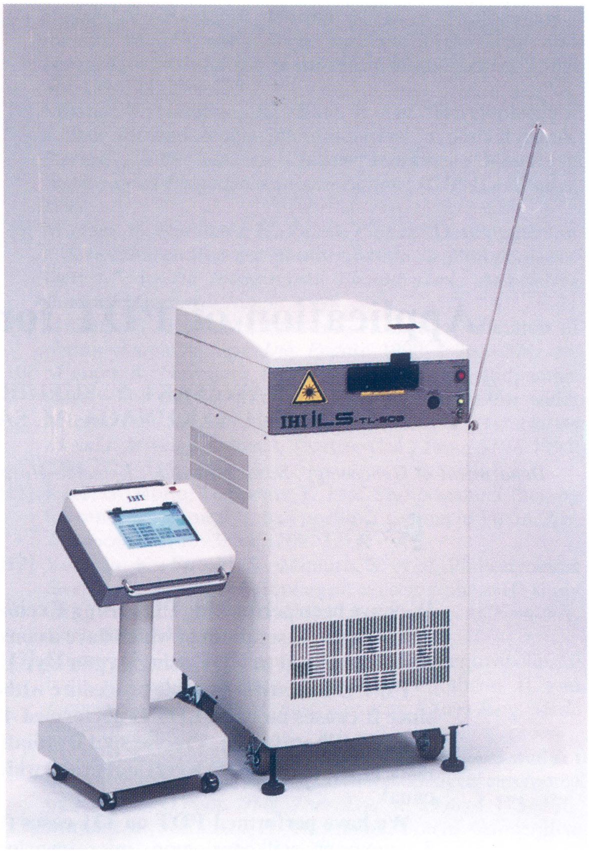

FIGURE 2 YAG-OPO laser.

of the cervical glands critical for reproduction tends to be lost during the operation.

To meet the demands of the increasing number of younger patients wanting to preserve fertility and to accommodate high risk patients, elderly patients, and those who refuse surgery, PDT has been used as a breakthrough method. PDT can be performed without anesthesia since there is no pain. Further, there is no bleeding with PDT, fertility can be preserved while leaving the cervix relatively intact, and there are no negative effects on pregnancy and delivery.

PDT, with the use of Excimer Dye Laser (EDL) (Fig. 1) a type of low power laser, has a considerably higher degree of tissue penetration, even compared to PDT using Argon Dye Laser (ADL). Also, PDT using EDL can manage glandular involvement of CIN, and its special feature of selective destruction of malignant cells with little effect on normal tissue is noteworthy. Beginning in 1995, PDT using YAG-OPO laser (Fig. 2) with a variable laser wavelength has been performed.

### MATERIALS AND METHODS

### Photosensitizer: PHE

Tumor affinity photosensitive substance: Porphyrin dimer to octomer mixed substance. Main component is dimer DHE (dehematoporphyrin ester/ether) a drug of freeze-dried powder consistency with a dark red color containing 75 mg PHE per vial (provided by Lederle (Japan), Ltd.).

### Laser Delivery Systems: EDL and YAG-OPO Laser

The EDL is a low energy power laser, and its 630 nm wavelength dye laser is generated when rhodamine 640 pigment solution is irradiated by 308 nm ultraviolet rays generated by an XeCl Excimer laser. The laser wavelength is 630–635 nm, width of pulse is $10 \pm 5$ nsec, and pulse radiation energy is 4–5 mJ/pulse maximum. Pulse repeated frequency is 40 Hz in normal cases (interchangeable to 40, 60, and 80 Hz) (Fig. 1).

YAG-OPO laser is a PDT laser that has an optic parametric oscillator (OPO), Q switch pulse YAG laser intensifier installed. Laser wave is 620–670 nm, pulse width is $7 \pm 1$ nsec, pulse irradiation energy is 6 mJ/pulse, and repeated frequency is 50 Hz (Fig. 2).

### Colposcope for Laser Therapy

A feature of the Olympus Laser Colposcope is an optical path for the laser and allows cervical lesions to be examined during photoirradiation. With this method it is possible to show a 10 mm circular spot at the focus where the observation is made. This results in stable and precise photoirradiation (Fig. 3).

### Cervical Probe

This probe was developed to administer PDT in the cervical canal, *i.e.*, endocervix. A special sapphire chip or ceramic chip is mounted on the tip of the cut fiber (Fig. 4).

### Laser Power Distribution of the Cervical Probe

It can administer photoirradiation in a forward direction on the cervical canal side walls: 70% of

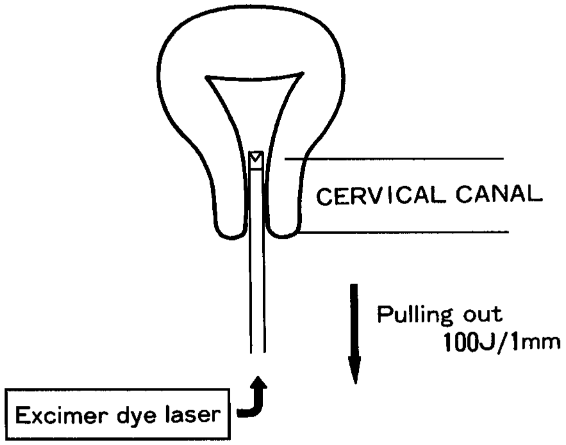

FIGURE 4 Cervical probe.

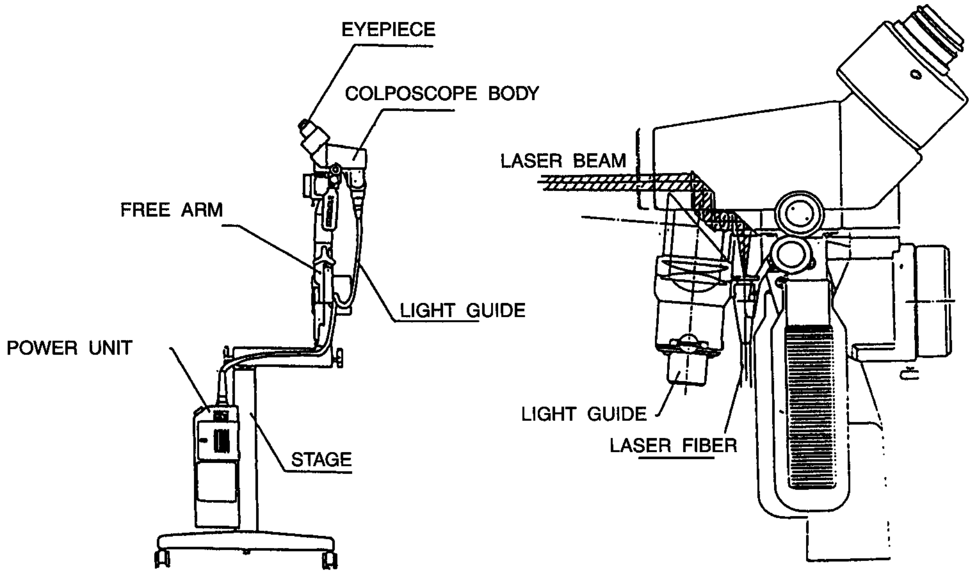

FIGURE 3 Colposcope for laser therapy.

the laser light is scattered to the side walls and 30% of the laser light forward. Thus, all of the cervical canal can be irradiated (Fig. 5).

### Cervical Probe Manipulator

Previously, the operator needed to mark the position where the cervical probe was originally inserted in order to withdraw in 1 mm increments. Presently, a cervical probe manipulator is installed in the colposcope, and it can move 0.5 mm in half revolutions and 1 mm in one complete revolution. This enables the operator to withdraw exactly 1 mm (Fig. 6).

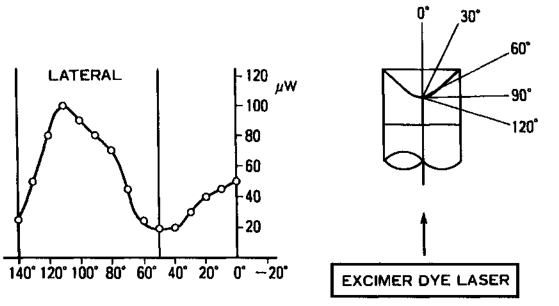

FIGURE 5 Laser power distribution of the cervical probe.

### Classification by Colposcopic Findings

According to the location of the lesions on the uterine cervix, the results of PDT are divided into the following three types:

- Type I means that the second S–C Junction was visible.
- Type II means that the second S–C Junction was not visible.
- Type III means Uncertifiable Colposcopic Findings.

As a rule, Type III cases were excluded from PDT because the irradiation would have been blind.

### Preparations Before Irradiation

1. Check patient's vital signs and confirm that patient has finished urinating.
2. Patient should wear a cloth to cover entire body and then proceed to the laser treatment room.

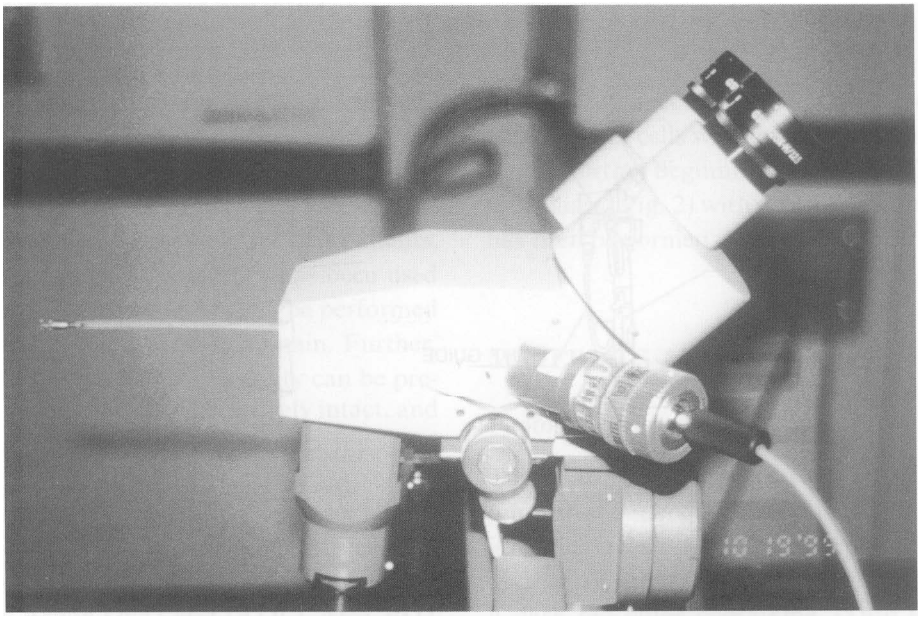

FIGURE 6 Cervical probe manipulator.

3. After putting on socks patient should get on table and then use paper tape to fix bath towels to minimize exposed areas.
4. Patient should cover genitals, other than the parts for irradiation by using gauze, and fix it by paper adhesive plaster.
5. Patient should wear sunglasses during the entire treatment period.
6. Cusco speculum should be inserted in a manner such that mobility is maintained, and fix the position slightly so as not to slide.
7. After sterilization of vagina using a disinfectant, acetic acid should be applied.

**Colposcope Irradiation (Spot Irradiation)**

1. Lesions located at the uterine cervix should be irradiated, particularly around the worst lesions at an energy intensity rate of 100 $J/cm^2$ per shot using an Olympus laser colposcope through direct observation.
2. A large amount of cervical mucous is produced by irradiation. It should be removed using swabs or a 1 cc syringe for cervical mucous extraction whenever needed.
3. Spot irradiation should overlap without leaving retention on the circumference. The pattern should resemble Olympic Game logo.
4. Typically parts, uneven surfaces and angles such as the cervical canal, should be irradiated in all directions by carefully adjusting the Cusco speculum and changing the laser angle. (Especially when glandular involvement exists, irradiation needs to be thorough as it tends to leave areas which have not been irradiated in such case.)

**Cervical Canal Irradiation**

After confirming the lesion is inside the cervical canal by using a hysteroscope, the cervical probe should be inserted to the required depth. Irradiation should be repeated as the inserted fiber is drawn from the cervical canal in 1 mm increments till it drops out from the external uterine orifice.

**Administration of Patients After PHE Injection**

Patients become extremely sensitive to sunshine for about a week after PHE injection. When exposed to intense light, the exposed skin reddens and may develop optic hyperesthesia such as edema. Therefore, the patient's room brightness should be measured using a lux meter and the following adjustments should be observed:

- Patient should stay in the room with a brightness of 10 lux immediately before the intravenous injection.
- After PHE injection, patient should stay in the room with a brightness of 10 lux for 4 days.
- From the 5th day after PHE injection, the intensity should be 15 lux, and patients should be allowed to watch television.
- From the 8th day after PHE injection, $\leq$ 30 lux.
- From the 11th day after PHE injection, $\leq$ 60 lux.
- From the 16th day after PHE injection, $\leq$ 80 lux.
- From the 19th day after PHE injection, $\leq$ 100 lux.
- From the 20th day after PHE injection, the restriction on the brightness is lifted and the patient may leave the hospital after sunset on that day.
- Patient should use UV (ultra violet rays) screen make-up cream and sun-shield cream as well as the administrated room brightness. When going out of the room, sunglasses, a hood, gloves, socks, a scarf, and a long sleeve dress should be worn so as not to be exposed to unnecessary light.

After leaving the hospital, patients are instructed to be cautious, avoiding direct sunshine for prolonged periods of time within two months after the PHE injection. Extreme caution should be observed when going outside at times when UV is stronger and when there is a lot of sunshine. Patients are also instructed to avoid intense direct sunshine for about six months after the PHE injection.

Also, it is understood that patients who have been exposed to sunlight from sunbathing before hospitalization are less likely to develop side effects than patients who have not done so.

### Evaluation of Effectiveness of PDT

Two months after PDT patients are checked by cytological, colposcopic, histopathological findings, and they are divided into the following four levels. Patients classified below PR are re-examined four months after PDT:

- CR: All lesions are completely cured in terms of cytological, colposcopic, and histopathological findings.
- PR: Almost all lesions are cured, but cytological, colposcopic, and histopathological findings suggest that some lesions may not yet be cured.
- NC: Most lesions are not cured, and cytological, colposcopic, and histopathological findings before PDT and after PDT are almost the same. Lesions from pre-PDT are completely preserved.
- PD: Among cytological, colposcopic, and histopathological findings, more than one finding became worse than before PDT.

### RESULTS

In our practice, 85% of all patients who received PDT wanted to preserve fertility. Only 1 patient had complications, 17 patients refused to undergo conventional surgery and 2 were elderly (Table I). Studies using the PDT method have been conducted on 131 patients (95 CIS, 31 dysplasia, and recently 3 squamous cell carcinoma, microinvasion (Fig. 7), and 1 CIS + endocervical adenocarcinoma, microinvasion, 1 vulval dysplasia). Out of 131 cases, 127 became CR (96.9%). In addition, all microinvasive cases became CR. Only 4 cases showed any remnants in a very limited area but most of the lesions had disappeared (Table II). One PR case became CR after a second PDT.

TABLE I Age and Reason for PDT

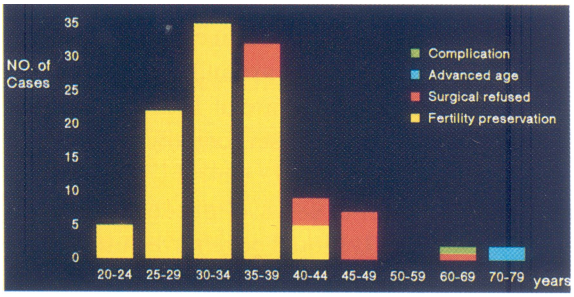

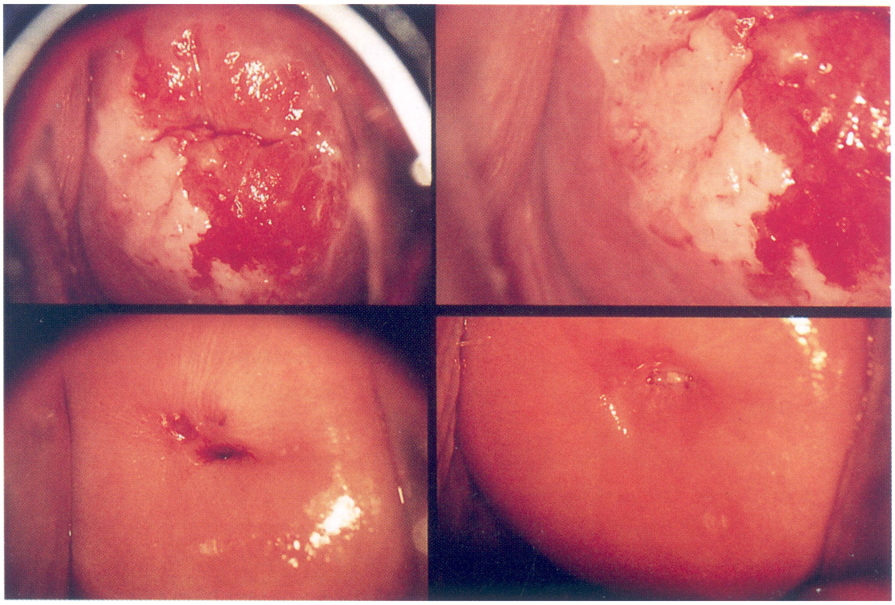

FIGURE 7 S.C.C. Ia case before and after PDT (32 year old patient). upper left: low-power view; upper right: high-power view; lower left: 11 weeks after PDT; lower right: 5 months after PDT.

TABLE II Response

| | Cases | CR | PR | NC |
|---|---|---|---|---|
| Dysplasia | 31 | **30(96.8%)** | 1(3.2%) | 0(0%) |
| CIS | 95+1 | **92+1(96.88%)** | 2(2.08%) | 1(1.04%) |
| S.C.C. Ia | 3 | **3(100%)** | 0(0%) | 0(0%) |
| EC-Ad-ca Ia | 1 | **1(100%)** | 0(0%) | 0(0%) |
| VIN* | 1 | **1(100%)** | 0(0%) | 0(0%) |
| Total | 131+1 | **127+1(96.97%)** | 3(2.27%) | 1(0.75%) |

*VIN (Vulvar Intraepitheliar Neoplasia).

Only 1 NC case would have been PR if the present standard was applied then. Lesions evenly spread on the vaginal wall were highly suspected to be invasive cancer. After receiving PDT the patient became pregnant and had a normal delivery. Subsequently, since remnants were found in the deep cervical canal, a semi-radical hysterectomy was performed. Histopathological findings on the extracted uteruses showed that the original lesions on the vaginal wall and in the cervical canal had almost disappeared, and the only remnant found was in the deep internal cervical canal, where it was suspected that the first PDT did not reach.

We found remnants only in the cervical canal in 1 PR case which was CIS. The remnants had disappeared and became CR after a second PDT was performed. Three and a half years have passed after the second PDT, and so far there has been no recurrence reported. One severe dysplasia and CIS cases were PR. Some remnants within a very limited area were seen after PDT and are still under observation.

The first PDT was administered about nine years ago. After PDT treatment 22 patients became pregnant, and out of those patients, 12 had normal transvaginal deliveries and 4 had cesarean sections (due to the following reasons obstetrical procedures were performed: one case had a cesarean section on the first delivery; another was premature at eight months; another case was CPD) and 3 are currently pregnant and 3 have had abortions. In 3 cases even a second child was born. They did not develop any adhesion or constriction of the cervical canal, and no difficulties were reported connected with childbirth after PDT. Also, even where there were stump remnants, in which a total hysterectomy is usually required after cold knife conization, the patient became CR after PDT and had a cesarean section (patient had previously given birth by cesarean section).

### Side Effects

Side effects of PDT are similar to symptoms of sunburn such as reddened skin due to sensitive reaction to sunlight. But only 20 cases among 131 required treatment, and they improved the following day after applying steroid ointment. In some cases, in the summer, when skin is exposed to sun, there was some incidence of skin irritation but not serious. Applying calamine lotion relieved the symptoms.

It is necessary to administer strict shading control in order to avoid side effects from sunburn, but as long as the shading time control is adhered to, there has been almost no problem reported. No side effects or abnormal findings have been recognized in blood and urine tests.

### DISCUSSION

One of the points which should be noted by gynecologists is the increase in the trend of younger patients who want to preserve fertility. About 85% of our PDT patients wanted to preserve fertility, and for that purpose preserving the uterus is critical. In order to respond to these patients' demands, PDT is well suited since it leaves the cervix relatively intact. PDT can be performed without anesthesia since there is no pain. In addition, there is no bleeding using PDT.

The recovery ratio after PDT is very high. No difficulties have been experienced during childbirth due to PDT, and further, transvaginal deliveries are possible. Also, no negative effect on pregnancy and the child have been reported. Such advantageous points have been receiving a lot of attention. The primary limitation of PDT is its similarity to symptoms of sunburn. Our PDT is administrated to relatively younger patients who are more concerned about skin blemishes and spots. Strict shading time should be observed by the patient. Insofar as this point is kept, we have seen no particular side effects as yet.

There are a number of points which can be cited as reasons for the favorable results of PDT. First, it is possible to obtain a relatively accurate diagnosis by cytological, colposcopic, and histopathological findings before the therapy is administered. Second, we use a pulse laser with a higher tissue penetration capability. Third, now it is possible to have stable and precise spot irradiation through direct observation using a colposcope equipped with an optical path for a laser. Fourth, for cervical canal treatment we use a cervical probe with the function to administer photoirradiation to the entire cervical canal. Thus, it became quite effective on glandular involvement cases and improved the total CR ratio.

Both EDL and PHE (Photofrin®) have received the approval of the Japanese Ministry of Health and Welfare, and since April 1996, patients have been covered by the Japanese national health insurance program. Also, clinical trials of lasers with variable laser wavelength (YAG-OPO; manufactured by IHI) have commenced, and a new type of diode laser has also been in the preparation stages for clinical trials. These latest developments should benefit and promise even more technological advancements of PDT in the future.

At the same time, due to the ongoing progress and development of different photosensitizers which have shorter retention time, shortening the shading and hospitalization time has been promised. First generation compounds, such as PHOTOFRIN®, require that the patient be hospitalized for a period from three weeks to two months and necessitate strict shading. In the future we expect that PDT can be performed without any hospitalization using new photosensitizers such as BPD-MA, ATX-S10, and NPe6.

### *References*

[1] Muroya, T., Sugishita, T., Tenjin, Y. *et al.* Clinical tests for phase III study of PDT for the early cervical cancer and pre-cervical cancer lesions by PHE (Porfimersodium) and eximer dye laser (PDT EDL-1). *Oncol Chemotherapy* 1992; **8**(3): 302–307.

[2] Muroya, T., Sugishita, T., Tenjin, Y. *et al.* Fertility preservation treatment for early cervical cancer and dysplasia by PDT (photodynamic therapy). *Oncol Chemotherapy* 1993; **9**(1): 21–32.

[3] Muroya, T., Sugishita, T., Tenjin, Y. *et al.* Photodynamic therapy (PDT) clinical trials. From the viewpoint of colposcopic, cytological, hysteroscopical changes. *J. Jpn. Soc. Laser. Med.* 1994; **15**(1): 41–52.

[4] Muroya, T., Sugishita, T., Tenjin, Y. *et al.* Photodynamic therapy for early cervical cancer. *Cancer Chemotherapy* 1996; **23**(1): 47–56.

[5] Muroya, T., Sugishita, T., Tenjin, Y. *et al.* Uterus preservation operation for CIN. PDT's clinical trial results. *J. Jpn. Soc. Obstet. Gynecol. Surg.* 1996; **7**: 27–38.

[6] Muroya, T. New strategies in cancer treatment–photodynamic therapy. *J. Tokyo. Med. Coll.* 1997; **55**(3): 408ff.

[7] Muroya, T., Sugishita, T., Tenjin, Y. *et al.* Our Method. PDT for CIN. *J. Obstet. Gynecol. Therapy.* 1998; **77**(4): 441–443.

